# Supplementary figures and images for: Absence of transmission of vYF next generation Yellow Fever vaccine in mosquitoes
Source: PLoS Negl Trop Dis. 2022 Dec 14;16(12):e0010930. doi: 10.1371/journal.pntd.0010930 (PMC9749985; doi:10.1371/journal.pntd.0010930)

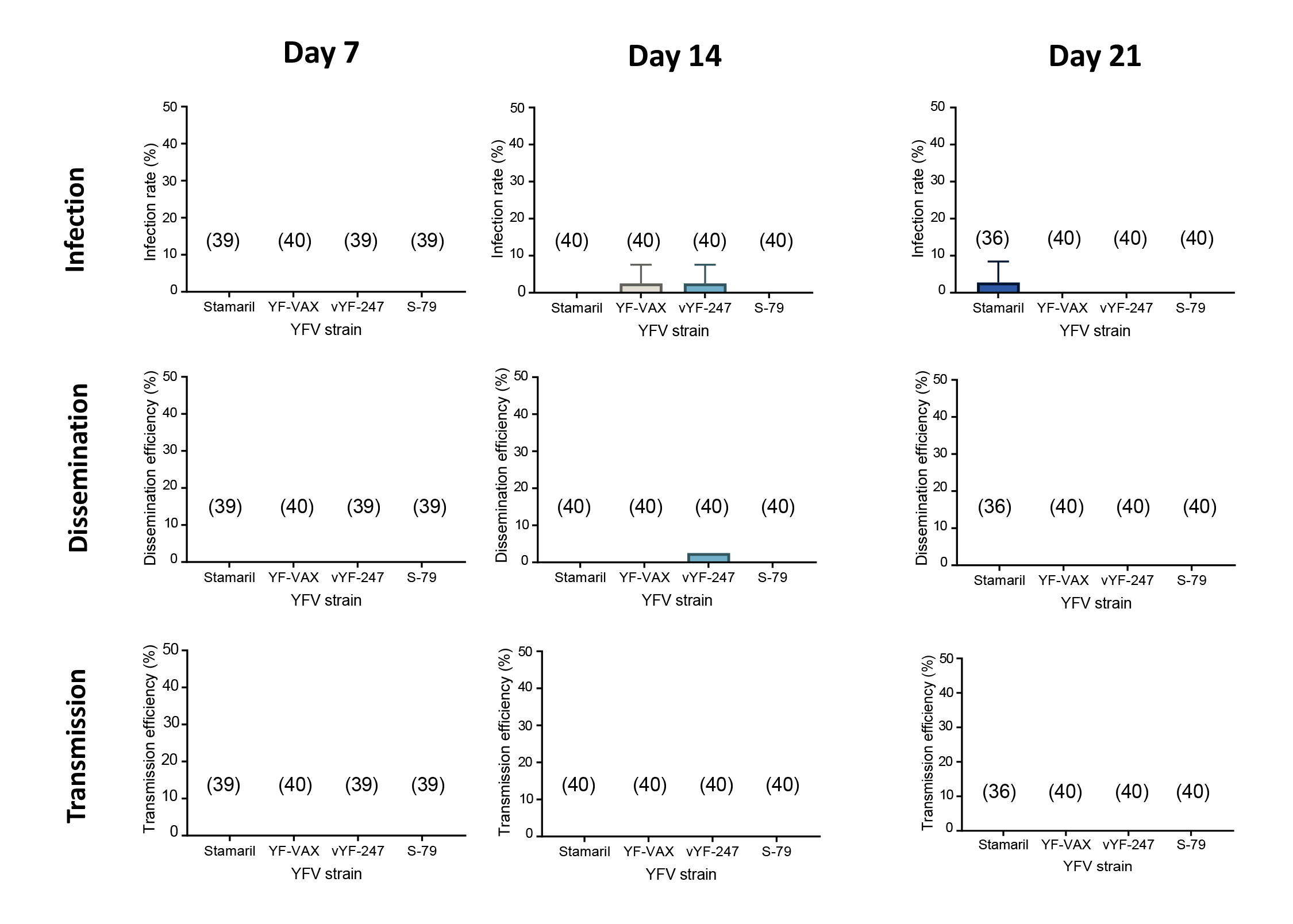

Supplement: S1 Fig — In brackets, number of mosquitoes. IR corresponds to the proportion of mosquitoes with an infected abdomen among tested mosquitoes. DE refers to the proportion of mosquitoes with infected HT among tested mosquitoes. TE is the proportion of mosquitoes with infectious saliva among tested mosquitoes. (TIF) [file pntd.0010930.s001.tif]

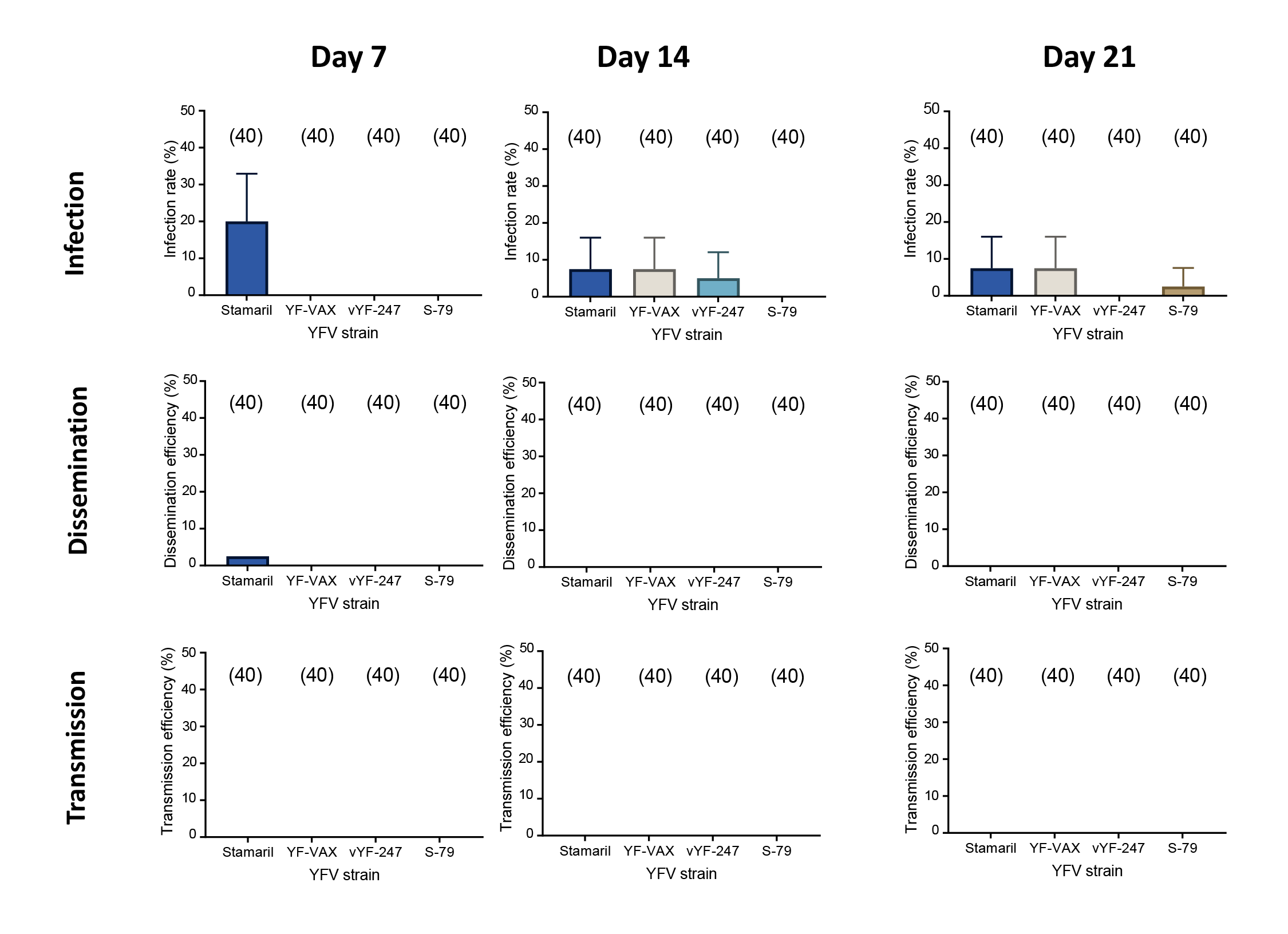

Supplement: S2 Fig — In brackets, number of mosquitoes. IR corresponds to the proportion of mosquitoes with an infected abdomen among tested mosquitoes. DE refers to the proportion of mosquitoes with infected HT among tested mosquitoes. TE is the proportion of mosquitoes with infectious saliva among tested mosquitoes. (TIF) [file pntd.0010930.s002.tif]
