# Supplementary material for: Absence of transmission of vYF next generation Yellow Fever vaccine in mosquitoes
Source: PLoS Negl Trop Dis. 2022 Dec 14;16(12):e0010930. doi: 10.1371/journal.pntd.0010930 (PMC9749985; doi:10.1371/journal.pntd.0010930)
Supplement: Attachment — Submitted filename: PNTD-D-22-00890.pdf [file pntd.0010930.s003.pdf]

In the article "Absence of transmission of vYF next generation Yellow Fever Vaccine in mosquitoes", Bellone and colleagues describe the results obtained by vector competence studies of *Ae. aegypti* and *Ae. albopictus* mosquitoes for vFY-247 vaccine compared to YF-17D-204 reference vaccines and a human isolate. This work corresponds to the first-time experimental infection in mosquitoes for vYF next generation yellow fever vaccine. The obtained results are relevant to develop new life attenuated vaccines following WHO's recommendations.

My review includes a few minor suggestions (text corrections or comments requested to the authors).

Lines 30: Please, change midgut by abdomen since the midgut was not dissected.

Lines 31: Please, eliminate "did not disseminate" since the authors observed dissemination (1/32) when Ae. aegypti colony (AAPAEA) and field-collected population from Taiwan Mid-West population (2/40) were exposed to vYF-247.

Line 66: Please, change the reference 1 by other reference more recent.

Lines 66-69: Please, add a reference.

Lines 79-80: Do all the references 11-13 fit well with the sentence: YFV-17D can infect *Ae. aegypti* midgut? Which(s) reference(s) support that YFV-17D does not disseminate to other internal tissues? In the discussion section (lines 308-309), it is related with reference 12, but could reference 13 be adequate? Please, add the adequate reference at the end of this sentence.

Line 93: Please, add the reference of this Annex 5.

Lines 93-95: Please, give more details on YF-17-204 and reference vaccines origins.

Line 109: Why do the authors rear the mosquitoes at 24ºC and 12:12 hour photoperiod.

Line 112: When, in terms of age, did the mosquitoes were exposed the viruses?

Lines 138-139: Why field-collected mosquitoes were only analyzed at 21 dpi?

Line 144: Please, specify in the serial dilutions were tenfold dilutions.

Line 149: Please, add "/mL" after "ffu".

Line 150: Please, specify if all tested mosquitoes were full engorged or blood-feed.

Lines 192-194: Please re-write this sentence. Statistics could only support this statement at 21 dpi for *Ae. aegypti* and with $p<0,01$ not 0,05.

Line 199: Please, add "tested" after "number of" if it is the case.

Fig. 1: Please modify the scale by indicating the same scale for all the graphics to be more comparable among them at the first glance.

Fig. 1, 3 and 4: Please, edit the y axis to give the proportions in % since the title of the axis is indicated in this way.

Line 222: Please, add “positive” after “number of” if it is the case.

Fig. 2, 3 and 4: Please, change viral particles by ffu/mL in the title of the figure and in the y axis.

Line 253: Delete one “of”.

Fig. 2, 3 and 4: Please, specify which numbers are indicated between brackets to be clearer, since they are different in the different kind of graphics.

Line 284: Please, give write “Eliminate yellow fever epidemics” before the abbreviation EYE if it is the case and provide the reference.

Lines 314-316: Since in the present study dissemination was found for vYFV-247 in *Ae. aegypti*, it should be pointed out and these statements should be modified to be less categorical.

Lines 320-321: It is true that the insecticide resistance is becoming a problem for vector control, but in case of the mosquito species involved in YFV transmission, the difficulty of insecticide treatment of the breeding sites of these species is also a problem for their control.

Lines 321-324: Please, add a (some) reference(s) for this sentence.

The scientists claim in the abstract that it is improbable that mosquitoes would transmit the vYF-247 vaccination given their findings and the undetectable viremia in vaccinees. All this information, including the reference utilized to claim that the vaccinees have minimal levels of viremia, should be mentioned in the discussion area as well.
