# Supplementary material for: Absence of transmission of vYF next generation Yellow Fever vaccine in mosquitoes
Source: PLoS Negl Trop Dis. 2022 Dec 14;16(12):e0010930. doi: 10.1371/journal.pntd.0010930 (PMC9749985; doi:10.1371/journal.pntd.0010930)
Supplement: Attachment — Submitted filename: MS Bellone_Answers to Reviewers_11 10 2022.pdf [file pntd.0010930.s005.pdf]

Dear Editor,
Thank you very much for considering our manuscript "Absence of transmission of vYF next generation Yellow Fever Vaccine in mosquitoes" for publication in PLOS Neglected Tropical Diseases.
Please find below the detailed list of our responses to the reviewer #2's comments, and the description of the changes we have made in the manuscript.

Sincerely yours,

Prof. Anna-Bella FAILLOUX
Institut Pasteur, Department of Virology
Arboviruses and Insect Vectors Unit
25 rue du Dr Roux,75724 Paris cedex 15

No questions from the Reviewer #1

Answers to Reviewer #2
Point #1: the paper states, "The WHO-TRS 978-Annex 5: Recommendations to assure the quality, safety and efficacy of live attenuated yellow fever vaccines recommends to assess mosquito infectivity and dissemination of any new YF vaccine candidates in comparison with a currently acceptable vaccine." Later in the paragraph the authors note that they test the vaccine strains "when feasible". This is a good qualifier that implies they weren't always able to compare the vaccine candidate to the current vaccines. However, I still think it is important that the authors state more clearly that the tests with the current vaccines did not yield significant results. Only the wt vs vaccine candidate tests yielded significant results. Therefore, further comparison to vaccine strains should be listed in the discussion as a future direction.
For future regulatory requirements, this paper alone should not be used to justify the approval of the vYF-247 vaccine based on mosquito transmissibility. According to WHO requirements, mosquito transmission of any YF vaccine candidate must be compared to current 17D vaccines. This paper does compare vYF to current 17D vaccines but the data is incomplete. Thank you for stating that viremia after vYF-247 vaccination is less than 106.5 ffu/mL; it adds good context to this issue. However, since no pattern or conclusion can be drawn from the 106.5 ffu/mL data, it's not fair to say that the comparison between vYF-247 and the current 17D vaccines is complete.

Answer: We understand your point and we have tried to state more clearly that the comparison with current vaccines did not yield significant results. Changes have been included in:

- Abstract (lines 31-34): "Although comparison of vYF-247 to reference vaccines could not be completed to yield significant results, we showed that vYF-247 was not transmitted by both *Aedes* species, either laboratory strains or field-collected populations, compared to clinical strain S-79 at the highest inoculation dose."
- Discussion (lines 308-314): "Indeed, results obtained from the comparison of the vYF-247 vaccine candidate to the reference vaccine at $10^{6.5}$ ffu/mL did not yield interpretable data since clinical strain S-79 was not either transmitted at this titer. The WHO-TRS 978-Annex 5: *Recommendations to assure the quality, safety and efficacy of live attenuated yellow fever vaccines* recommends to assess mosquito infectivity and dissemination of any new YF vaccine candidates in comparison with a currently

acceptable vaccine (21). Thus, further comparisons of vYF-247 vaccine candidate to the reference vaccines will be required to complete this work.”

Point #2: I appreciate that the authors changed some of the wording for the comparison between vYF-247 and the vaccine references (e.g., "when feasible"). This makes the analysis presented more clearly represent the analysis plan.
In the experiments that include the YF vaccine references, no real correlation can be seen between vYF-247 and the vaccines, between vYF and the wild type, or between the wild type and the reference vaccines. With this in mind, it is not possible to draw conclusions from the vaccine references (data in the supplemental figures) to the vaccine candidate (data in the main paper).
The authors analyze each mosquito section and mosquito population as intended. They also compare each population tested as intended.
The figures are clear and concise and show all relevant information. The error bars, lines indicating averages, and significance bars are clear. None of the figures are cluttered. The mosquito diagram in figure 1 is particularly helpful to understand the paper. The X and Y axis labels may be a little small but can be easily read on a digital copy. It is easy to distinguish between the colors in each figure.
The results are presented clearly and linearly. They also appear to be completely presented and tell a good story from start to finish.

Answer: Regarding correlation in the experiments that include the YF vaccine references, we are aware that this represents a major limitation in our study. We have addressed the issue in the discussion.

Point #3: The first sentence of the discussion is the main conclusion of this paper and it is supported by the data presented. The conclusions are not overstated and the authors do a good job not to mention comparison to reference vaccines in their statement of the main conclusions. The data collected are easy to understand and the conclusion drawn is clear.
The major limitation of this paper (not being able to compare vYF-247 to the vaccine references) is stated and explained well in the discussion. Their reasoning is understandable and all too relatable. Thank you for changing the wording related to this in the abstract and introduction. However, I believe the wording in the author summary should be changed slightly. On line 44, "but also" is slightly awkward and indicates the reference vaccines were tested to the same degree as vYF-247 and S-79. To make this more clear, it would be beneficial to say something like, "vYF-247 was primarily compared to a clinical isolate and secondarily to reference vaccines, though no significant conclusions could be drawn from the comparisons to the reference vaccines." It would be useful to have a statement like this in the body of the paper as well so readers do not misinterpret the findings of the paper.

Answer: As recommended, we have modified the author summary in lines 44-50: “Here we studied the infection, dissemination and transmission of vYF-247 vaccine candidate compared primarily to an YF clinical isolate S-79 and secondarily to two reference Sanofi vaccines (Stamaril and YF-VAX) in mosquitoes, *Aedes aegypti* and *Aedes albopictus*. Although no significant conclusions could be drawn from the comparisons to the reference vaccines, we nevertheless showed that vYF-247 was unable to be transmitted by mosquitoes at the highest inoculation dose.”

Point #4: Line 33: Saying that vYF-247 was not transmitted by mosquitoes at "both tested titers" is technically correct but may be slightly misleading. It is important to say what vYF-247 is being compared to in each case. The data the authors have collected in comparison to

the wild type at the higher titer is good data! The data for the lower titer is not as good, so saying "both tested titers" here gives the wrong impression.

Answer: The wording has been corrected in the abstract in lines 31-34: “Although comparison of vYF-247 to reference vaccines could not be completed to yield significant results, we showed that vYF-247 was not transmitted by both *Aedes* species, either laboratory strains or field-collected populations, compared to clinical strain S-79 at the highest inoculation dose.”

Point #5: Line 67: change from "Spillovers were." to either "Spillovers are." or "Spillovers can be."
Answer: Thanks

Point #6: Line 91-94: As noted above, the authors may want to move this to the discussion section to be used as a future direction.
Answer: The sentence has been moved to discussion section in lines 306-312: “Indeed, results obtained from the comparison of the vYF-247 vaccine candidate to the reference vaccine at $10^{6.5}$ ffu/mL did not yield interpretable data since clinical strain S-79 was not either transmitted at this titer. The WHO-TRS 978-Annex 5: *Recommendations to assure the quality, safety and efficacy of live attenuated yellow fever vaccines* recommends to assess mosquito infectivity and dissemination of any new YF vaccine candidates in comparison with a currently acceptable vaccine (21). Thus, further comparisons of vYF-247 vaccine candidate to the reference vaccines will be required to complete this work.”

Point #7: Apologies if I was unclear about the use of semicolons in the previous revisions!
Lines 172-175: Recommend use of semicolons. "ALPROV was able to become infected at 7 dpi with Stamaril (20%; 8/40); at 14 dpi with Stamaril (7.5%; 3/40), YF-VAX (7.5%; 3/40), and vYF-247 (5%; 2/40); and at 21 dpi with Stamaril (7.5%; 3/40), YF-VAX (7.5%; 3/40), and S-79 (2.5%; 1/40)."
Answer: the changes have been made.

Point #8: Lines 210-212: for clarity, add semicolons so the section reads like this: "103.2 at 7 dpi, 103.5 at 14 dpi, and 103.3 at 21 dpi in abdomens; 102.6 at 7 dpi, 105.0 at 14 dpi, and 105.5 at 21 dpi in HT; and at 21 dpi, an average of 101.8 particles was detected in saliva."
Answer: the changes have been made.

Point #9: Lines 247-253, 268: may want to add "ffu" after the numbers for clarity on how viral particles are being measured.
Answer: we have added ffu.

Point #10: Line 296: Could start a new paragraph with "At the outset." since it is the start of a new topic. May also want to clarify by saying, "At the outset of this study." to tell the reader that you are no longer talking about clinical trials, but that you are talking about your current mosquito study.
Answer: the change has been made.
