# Supplementary material for: Absence of transmission of vYF next generation Yellow Fever vaccine in mosquitoes
Source: PLoS Negl Trop Dis. 2022 Dec 14;16(12):e0010930. doi: 10.1371/journal.pntd.0010930 (PMC9749985; doi:10.1371/journal.pntd.0010930)
Supplement: Attachment — Submitted filename: MS Bellone_Answers to Reviewers.docx [file pntd.0010930.s004.docx]

Dear Editor,

We made most changes suggested. Please, find below point-by-point answers to the reviewers, indicating which changes have been made, and where they have been inserted in the text (highlighted in yellow in the revised version).

Sincerely yours,

**Prof. Anna-Bella FAILLOUX**

Institut Pasteur, Department of Virology

Arboviruses and Insect Vectors Unit

25 rue du Dr Roux,75724 Paris cedex 15

**Answers to Reviewer #1**

My review includes a few minor suggestions (text corrections or comments requested to the authors).

**Point #1: Lines 30: Please, change midgut by abdomen since the midgut was not dissected.**

It has been corrected.

**Point #2: Lines 31: Please, eliminate “did not disseminate” since the authors observed dissemination (1/32) when Ae. aegypti colony (AAPAEA) and field-collected population from Taiwan Mid-West population (2/40) were exposed to vYF-247.**

Dissemination of vYF-247 occurs even very rarely. We only state: “vYF-247 was not transmitted by both *Aedes* species”.

**Point #3: Line 66: Please, change the reference 1 by other reference more recent.**

We have replaced by: Gianchecchi E, Cianchi V, Torelli A, Montomoli E. Yellow Fever: Origin, Epidemiology, Preventive Strategies and Future Prospects. Vaccines (Basel). 2022 Feb 27;10(3):372. doi: 10.3390/vaccines10030372.”

**Point #4: Lines 66-69: Please, add a reference.**

We added the reference of Barrett & Higgs (2007): “Yellow fever: a disease that has yet to be conquered” (Annu Rev Entomol. 2007;52:209-29. doi: 10.1146/annurev.ento.52.110405.091454) gives a good description of YF cycle in Figure 1 of their review.

**Point #5: Lines 79-80: Do all the references 11-13 fit well with the sentence: YFV-17D can infect Ae. aegypti midgut? Which(s) reference(s) support that YFV-17D does not disseminate to other internal tissues? In the discussion section (lines 308-309), it is related with reference 12, but could reference 13 be adequate? Please, add the adequate reference at the end of this sentence.**

The references 11 and 12 demonstrated using infectious clones that substitutions of domain III of the envelope structural protein gene affect YFV on dissemination from the mosquito midgut.

We keep the reference 13 of Danet et al. (2019) where the authors demonstrated that YFV-17D replicated in the midgut of *Aedes aegypti* but failed to disseminate in the mosquito general cavity. We also used the reference Danet et al. (2019) in the discussion section.

**Point #6: Line 93: Please, add the reference of this Annex 5.**

The reference was inserted: World Health Organization. Recommendations to assure the quality, safety and efficacy of live attenuated yellow fever vaccines. WHO Technical Report Series No. 978. Replacement of Annex 2 of WHO Technical Report Series, No. 872 and of the Amendment to that annex in WHO Technical Report Series, No. 964 (2012). [Recommendations to assure the quality, safety and efficacy of live attenuated yellow fever vaccines, Annex 5, TRS No 978 (who.int)](https://www.who.int/publications/m/item/yellow-fever-vaccines-live-attenuated-annex-5-trs-no-978)

**Point #7:** **Lines 93-95: Please, give more details on YF-17D-204 and reference vaccines origins.**

Reference vaccines used in this study were Stamaril and YF-VAX commercial batch drug substance produced by Sanofi and amplified by one passage on Vero cells. This was mentioned in the manuscript. Both vaccines were derived from YF-17D-204 substrain as described in the diagram below (Fig 2. from reference 15)

**Point #8: Line 109: Why do the authors rear the mosquitoes at 24ºC and 12:12 hour photoperiod.**

These conditions reflect a compromise between tropical mosquitoes and temperate mosquitoes. Tropical mosquitoes can be reared at a temperature > 24°C and temperate mosquitoes at < 24°C.

At high temperatures, we faced high mortalities and at low temperatures, a delay in larval development.

**Point #9: Line 112: When, in terms of age, did the mosquitoes were exposed the viruses?**

It has been stated on line 131 “one-week-old female adults”.

**Point #10: Lines 138-139: Why field-collected mosquitoes were only analyzed at 21 dpi?**

Our first assessment using lab colonies showed that we are more likely to detect transmission of YFV at 21 days post-infection.

**Point #11: Line 144: Please, specify in the serial dilutions were tenfold dilutions.**

We have modified by “ten-fold dilutions.”

**Point #12: Line 149: Please, add “/mL” after “ffu”.**

It is the number of viral particles per organ: abdomen, head+thorax and saliva.

**Point #13: Line 150: Please, specify if all tested mosquitoes were full engorged or blood-feed.**

We have rephrased on lines 134-137: “Only fully engorged mosquitoes were kept and maintained in containers placed in climatic chambers at 28°C ± 0·1°C until processing at different days post-infection (dpi). Mosquitoes were fed with 10% sucrose solution.”

**Point #14: Lines 192-194: Please re-write this sentence. Statistics could only support this statement at 21 dpi for Ae. aegypti and with p<0,01 not 0,05.**

As suggested, we have replaced on lines 195-197 by: “We confirmed that the vaccine candidate vYF-247 was not transmitted by *Ae. aegypti* compared to the YFV S-79 (Fisher’s exact test: p < 0.01) and neither vYF-247 nor YFV- S-79 were transmitted by *Ae. albopictus*.”

**Point #15: Line 199: Please, add “tested” after “number of” if it is the case.**

We have modified accordingly.

**Point #16: Fig. 1: Please modify the scale by indicating the same scale for all the graphics to be more comparable among them at the first glance.**

As suggested, Figure 1 has been modified.

**Point #17: Fig. 1, 3 and 4: Please, edit the y axis to give the proportions in % since the title of the axis is indicated in this way.**

We made the changes as requested in Figures 1, 3, 4 and Figures S1, S2..

**Point #18: Line 222: Please, add “positive” after “number of” if it is the case.**

We have added “YFV-positive“.

**Point #19: Fig. 2, 3 and 4: Please, change viral particles by ffu/mL in the title of the figure and in the y axis.**

The number of viral particles has been estimated for each organ examined.

**Point #20: Line 253: Delete one “of”.**

Thanks

**Point #21: Fig. 2, 3 and 4: Please, specify which numbers are indicated between brackets to be clearer, since they are different in the different kind of graphics.**

We have modified accordingly:

In figure 1: In brackets, number of tested mosquitoes

In figure 2: In brackets, number of YFV-positive mosquitoes.

In figure 3: In brackets, number of tested mosquitoes for IR, DE, TE, and number of viral particles in abdomen, HT and saliva.

In figure 4: In brackets, number of tested mosquitoes for IR, DE, TE, and number of viral particles in abdomen, HT and saliva.

**Point #22: Line 284: Please, give write “Eliminate yellow fever epidemics” before the abbreviation EYE if it is the case and provide the reference.**

Thanks.

**Point #23: Lines 314-316: Since in the present study dissemination was found for vYFV-247 in Ae. aegypti, it should be pointed out and these statements should be modified to be less categorical.**

We have reformulated the sentence on lines 323-324: “As vYF-247 derives from YFV-17D, it is probable that both vaccines share some properties (25).”

**Point #24: Lines 320-321: It is true that the insecticide resistance is becoming a problem for vector control, but in case of the mosquito species involved in YFV transmission, the difficulty of insecticide treatment of the breeding sites of these species is also a problem for their control.**

We have added the following statement: “and presence of cryptic breeding sites which are difficult to implement vector control (29).”

Ong, J., Liu, X., Rajarethinam, J. *et al.* A novel entomological index, *Aedes aegypti* Breeding Percentage, reveals the geographical spread of the dengue vector in Singapore and serves as a spatial risk indicator for dengue. *Parasites Vectors* **12**, 17 (2019). <https://doi.org/10.1186/s13071-018-3281-y>

**Point #25: Lines 321-324: Please, add a (some) reference(s) for this sentence.**

We have added two references: “The increased number of YF-infected travelers despite the International Health Regulations, poor control of YF vaccination at entry to some countries and falsified vaccine certificates, increase the risk of introducing YF into new areas (30, 31).

Schonenberger S, Hatz C, Buhler S. Unpredictable checks of yellow fever vaccination certificates upon arrival in Tanzania. J Travel Med 2016; 23: 5.

Monath TP, Gershman M, Hill DR, Marano N, Staples JE, Wilder-Smith A. Yellow fever recommendations for tourists to Kenya: a flawed risk

assessment. J Travel Med 2009; 16: 146.

**Point #26: The scientists claim in the abstract that it is improbable that mosquitoes would transmit the vYF-247 vaccination given their findings and the undetectable viremia in vaccinees. All this information, including the reference utilized to claim that the vaccinees have minimal levels of viremia, should be mentioned in the discussion area as well.**

We have added at the end of the discussion on lines 343-345: “Moreover, the absence of transmission by the two *Aedes* species combined with the low viremia developed by vaccinated people support that vYF-247 is a good vaccine candidate.”

**Answers to Reviewer #2**

**Point #1: Here, we tested the replication of the vaccine candidate vYF- 247 in mosquitoes, Ae. aegypti and Ae. albopictus in comparison with the YF-17D-204** **reference vaccines (Sanofi Stamaril and YF-VAX).” The study design for the majority of the paper only includes vYF and wild type YF, not the vaccine references.** **The part of the study design that includes the vaccine references does not have strong data and doesn’t imply a correlation between vYF-247 and the vaccines, between vYF and the wild type, or between the wild type and the reference vaccines. Without any of these correlations, the vYF vaccine candidate cannot be compared to the vaccine references as the authors state in the quote I mentioned above.**

We agree that it is difficult to draw conclusions when comparing the performances of the 4 viral strains provided at 10^6.5^ FFU/mL and vYF-247 with S-79 at 10^7.5^ FFU/mL. It mainly demonstrates that at a viral titer close to what can be detected in non-human primates and humans, transmission of vaccine candidate vYF-247 by mosquitoes as well as reference vaccines is very unlikely.

The sentence has been changed to better match with the experimental design. See on lines 25-29: “Here we infected *Aedes aegypti* and *Aedes albopictus* mosquitoes with vYF-247 vaccine compared first to the YF-17D-204 reference Sanofi vaccines (Stamaril^®^ and YF-VAX^®^) and a clinical human isolate S-79, provided in a blood meal at a titer of 6.5 Log ffu/mL and secondly, to the clinical isolate only at an increased titer of 7.5 Log ffu/mL.”

**Point #2: The authors should consider doing these experiments in Haemagogus mosquitoes as well, as these mosquitoes can transmit YF in South America. Otherwise, the populations are clearly described and are appropriate for the hypothesis, which is to assess transmissibility from mosquitoes of vYF-247. Again, vYF-247 is not compared to the 17D reference vaccines, therefore, any hypothesis regarding comparison between these is not tested by these experiments.**

Although it would be very interesting to evaluate the replication of the vaccine candidate in Haemagogus mosquitoes (involved in a sylvatic cycle), we chose to focus on human-biting mosquitoes intervening in the rural/urban cycles which are more likely to bite vaccinated people. The comparison of vYF-247 and 17D reference vaccines is made in *Ae. aegypti* Paea and *Ae. albopictus* Providence at 10^6.5^ ffu/mL because we could not produce these reference vaccines at higher titers. At 10^6.5^ ffu/mL, neither the vYF-247 vaccine candidate nor the 17D reference vaccine is transmitted by both mosquito colonies.

**Point #3: There is a concern for future regulatory requirements. This paper alone cannot be used to justify the approval of the vYF-247 vaccine based on mosquito transmissibility. According to WHO requirements, mosquito transmission of any YF vaccine candidate must be compared to current 17D vaccines, which this paper does not adequately cover.**

The comparison between vYF-247 vaccine candidate and the current 17D vaccines was done for two mosquito lab colonies (data showed in supplementary data). WHO requires to evaluate the transmission capacity of the vaccine candidate in comparison to a currently acceptable vaccine by at least one mosquito strain. We did infections of two mosquito species: Paea and Providence. In order to give more weight to our results, we decided to also test mosquito populations infected with the vaccine candidate.

**Point #4: Everything in the analysis presented matches the analysis plan except for the comparison between vYF-247 and the vaccine references. In the abstract, the authors state that vYF-247 will be compared to 17D-204 reference vaccines and wild type virus. As the bulk of this paper discusses only a comparison between vYF-247 and the wild type, that statement is not accurate. The wording should be made clearer earlier in the paper (especially in the abstract and introduction) that the results only really compare the vaccine candidate to the wild type, and that there are very limited comparisons to the vaccine strains.**

**In the experiments that include the YF vaccine references, no real correlation can be seen between vYF-247 and the vaccines, between vYF and the wild type, or between the wild type and the reference vaccines. With this in mind, it is not possible to draw conclusions from the vaccine references (data in the supplemental figures) to the vaccine candidate (data in the main paper).**

The wording has been modified in abstracts and introduction. See lines 25-29: “Here we infected *Aedes aegypti* and *Aedes albopictus* mosquitoes with vYF-247 vaccine compared first to the YF-17D-204 reference Sanofi vaccines (Stamaril^®^ and YF-VAX^®^) and a clinical human isolate S-79, provided in a blood meal at a titer of 6.5 Log ffu/mL and secondly to the clinical isolate only at an increased titer of 7.5 Log ffu/mL.” and lines 94-97: “Here, we tested the replication of the vaccine candidate vYF-247 in mosquitoes, *Ae. aegypti* and *Ae. albopictus* in comparison with the clinical isolate S-79 but also the YF-17D-204 reference vaccines (Sanofi Stamaril and YF-VAX vaccine drug substance amplified by one passage on Vero cells) when feasible.”

**Point #5: The major limitation of this paper (not being able to compare vYF-247 to the vaccine references) is stated and explained well in the discussion. Their reasoning is understandable and all too relatable. However, this limitation is stated less clearly in the body of the paper and the abstract. The body and abstract should be changed to state that vYF-247 was only successfully compared to the wild type. Any mention of comparison between vYF-247 and the vaccine references should clearly state that this was not fully tested in this paper. Non-inferiority studies are important for the development of next-generation YF vaccines and these studies in mosquitoes should be done with the reference vaccines.**

We agree. The wording has been modified in the abstract, author summary and introduction.

- **Abstract on lines 25-29**: “Here we infected *Aedes aegypti* and *Aedes albopictus* mosquitoes with vYF-247 vaccine compared first to the YF-17D-204 reference Sanofi vaccines (Stamaril^®^ and YF-VAX^®^) and a clinical human isolate S-79, provided in a blood meal at a titer of 6.5 Log ffu/mL and secondly, to the clinical isolate only at an increased titer of 7.5 Log ffu/mL.”
- **Author summary on lines 42-45**: “Here we studied the infection, dissemination and transmission of vYF-247 vaccine candidate compared to an YF clinical isolate S-79 but also to two reference Sanofi vaccines (Stamaril^®^ and YF-VAX^®^) in mosquitoes, *Aedes aegypti* and *Aedes albopictus*.*”*
- **Introduction on lines 94-97**: “Here, we tested the replication of the vaccine candidate vYF-247 in mosquitoes, *Ae. aegypti* and *Ae. albopictus* in comparison with the clinical isolate S-79 but also the YF-17D-204 reference vaccines (Sanofi Stamaril and YF-VAX vaccine drug substance amplified by one passage on Vero cells) when feasible.”

The vYF-247 has been compared with the vaccine references provided to two lab-colonies at a titer of 10^6.5^ ffu/mL, higher than the viremia generated after vYF-247 vaccination of non-human primates or humans.

**Point #6:** **While not necessarily a limitation of this paper, it would be interesting to discuss transmission studies from mosquitoes to mammals with the viral strains used in this paper as a future direction for research.**

The reference vaccines (Stamaril^®^ and YF-VAX^®^) and the vaccine candidate vYF-247 candidate cannot disseminate in the mosquito cavity to be transmitted via mosquito bite. Therefore it would be difficult to study the transmission from infected mosquitoes to mammals.

**Point #7: Recommend major revisions for one reason: vYF-247 needs to be adequately compared to the reference vaccines. Either the experiments need to be optimized to show clear data for the reference vaccines or much of the wording of the paper needs to be changed to reflect the fact that vYF-247 is only successfully compared to wild type YF. Examples of lines that need to be changed: lines 25-28, 42-45, 90-95**

The wording has been modified in the abstract, author summary and introduction. See answers to point #5.

**Point #8: Regarding the discussion on viremia of vYF-247 in animal models (line 299-307): To make this point more clear, it would be useful to say something like, “****low viremia in these animal models combined with the mosquitoes’ inability to transmit vYF-247 even at the high titers illustrated in this paper indicates that transmission of vYF through a mosquito bite is highly unlikely.” As the paper is currently written, an untrained reader may not understand why the low viremia in animal models is mentioned in the discussion. The way it is stated in the abstract is closer to what I am proposing.**

The sentence has been modified on lines 313-315: “Therefore, the low viremia induced after vaccination combined with the mosquitoes’ inability to transmit vYF-247 indicates that transmission of vYF-247 through a mosquito bite is highly unlikely.”

**Point #9: Since viral diversity is mentioned in the discussion, it would also be worthwhile to mention that future studies of vYF-247 should include viral population diversity analysis as another measure showing its non-inferiority to** **current YF vaccines.**

The analysis of viral population diversity of vYF-247 vaccine candidate was described in Esson et al. Vaccine 2022.

We have added on lines 323-324: “As vYF-247 derives from YFV-17D, it is probable that both vaccines share some properties (25).”

**Point #10: Some of the results paragraphs get a little confusing when percentages and titers are listed one after the other. It may be worthwhile to use semicolons instead of commas to better delineate the data points that are being compared. Example: lines 169-173.**

We have replaced comas by semicolons.

**Point #11:** **Line 22: Insert the word “a” before “state-of-the-art”**

Done.

**Point #12:** **Line 54: change “originated” to “originating”**

Thanks.

**Point #13: Line 55: delete “with”. It is redundant after the word “alongside”.**

Thanks

**Point #14: Line 60: This sentence reads awkwardly. Maybe try putting the word “the” before “control” so it reads, “mosquito eradication campaigns of the 1940s and 1950s led to the control of YF…”**

Done.

**Point #15: Line 74: This would be better described as “epidemic” rather than “pandemic”.**

The change has been made.

**Point #16:** **Line 77: mouse tissue was used as well.**

Added.

**Point #17:** **Line 180: This sentence reads awkwardly. Try changing it to “****infection was only detected at 7 dpi” instead of “there were only infection detected at 7dpi**.”

The change has been made.

**Point #18:** **Line 241: swap the words “transmitted” and “successfully”**

The change has been made.

**Point #19:** **Line 279: Are the authors saying that the imported cases into China are examples of insufficient vaccination coverage because they should have been vaccinated as travelers to an endemic area? If so, that should be stated.**

We have added on line 285: “the imported cases of YF into China due to unvaccinated travelers returning from endemic areas”.

**Point #20:** **Line 281: 80% of what?**

We have replaced by: “at least 80% of vaccine coverage”

**Point #21:** **Line 284: Please spell out the whole name for EYE before using it as an acronym.**

It has been done.

**Point #22:** **Line 286-287: I disagree that the growth of the current YF vaccine in eggs is “the” major blocking point. It would be more realistic to say that it is “a” major blocking point.**

It has been modified as requested.

**Point #23:** **Line 310: Check the spelling on “envelope”.**

Thanks.
